# Identification of *Pseudomonas aeruginosa* Phenazines that Kill *Caenorhabditis elegans*

**DOI:** 10.1371/journal.ppat.1003101

**Published:** 2013-01-03

**Authors:** Brent Cezairliyan, Nawaporn Vinayavekhin, Daniel Grenfell-Lee, Grace J. Yuen, Alan Saghatelian, Frederick M. Ausubel

**Affiliations:** 1 Department of Genetics, Harvard Medical School, Boston, Massachusetts, United States of America; 2 Department of Molecular Biology, Massachusetts General Hospital, Boston, Massachusetts, United States of America; 3 Department of Chemistry and Chemical Biology, Harvard University, Cambridge, Massachusetts, United States of America; 4 Program in Immunology, Harvard Medical School, Boston, Massachusetts, United States of America; Stanford University, United States of America

## Abstract

Pathogenic microbes employ a variety of methods to overcome host defenses, including the production and dispersal of molecules that are toxic to their hosts. *Pseudomonas aeruginosa*, a Gram-negative bacterium, is a pathogen of a diverse variety of hosts including mammals and the nematode *Caenorhabditis elegans*. In this study, we identify three small molecules in the phenazine class that are produced by *P. aeruginosa* strain PA14 that are toxic to *C. elegans*. We demonstrate that 1-hydroxyphenazine, phenazine-1-carboxylic acid, and pyocyanin are capable of killing nematodes in a matter of hours. 1-hydroxyphenazine is toxic over a wide pH range, whereas the toxicities of phenazine-1-carboxylic acid and pyocyanin are pH-dependent at non-overlapping pH ranges. We found that acidification of the growth medium by PA14 activates the toxicity of phenazine-1-carboxylic acid, which is the primary toxic agent towards *C. elegans* in our assay. Pyocyanin is not toxic under acidic conditions and 1-hydroxyphenazine is produced at concentrations too low to kill *C. elegans*. These results suggest a role for phenazine-1-carboxylic acid in mammalian pathogenesis because PA14 mutants deficient in phenazine production have been shown to be defective in pathogenesis in mice. More generally, these data demonstrate how diversity within a class of metabolites could affect bacterial toxicity in different environmental niches.

## Author Summary

The bacterium *Pseudomonas aeruginosa* is a pathogen of a wide variety of organisms. It has been shown that *P. aeruginosa* factors that are critical for its toxicity to the nematode worm *Caenorhabditis elegans* are also important for its pathogenicity in mammals. In this report we show that phenazines, a class of small molecules produced by *P. aeruginosa*, act as lethal toxins against the worm. Under conditions relevant to mammalian pathogenesis, we identified one phenazine, phenazine-1-carboxylic acid, that is primarily responsible for killing worms. We found that the toxicity of this phenazine and one other phenazine, pyocyanin, are dependent on the pH of the media. We also identified a third toxic phenazine, 1-hydroxyphenazine, whose toxicity is not dependent on pH. These results show that the diversity of toxic molecules produced and released by *P. aeruginosa* may serve the bacterium to facilitate pathogenicity in a variety of environments.

## Introduction

The Gram-negative bacterium *Pseudomonas aeruginosa*, a pathogen of both plants and metazoans, is a prevalent and pernicious pathogen in persons who are immunocompromised or suffer from cystic fibrosis (CF) [1], [2]. *P. aeruginosa* employs many mechanisms to antagonize its hosts, including the production of low molecular weight toxins [3], [4], [5]. Identifying toxins, the conditions under which they are produced, and the mechanisms by which they act, are of fundamental importance in understanding and combating the virulence of this clinically-important pathogen.

The nematode *Caenorhabditis elegans*, which is found in decaying plants where many pathogenic microbes reside, is a useful model host for a variety of pathogens including *P. aeruginosa* [6], [7]. PA14 is a virulent clinical isolate of *P. aeruginosa* that is capable of killing *C. elegans* [8], [9]. Previous studies using *C. elegans* as a model system for PA14 pathogenesis determined that PA14 can kill *C. elegans* either as a consequence of intestinal infection or intoxication, depending on the media on which the bacteria are grown [8], [9]. Nematodes die in hours when PA14 is grown on a nutrient rich agar containing peptone, glucose, and sorbitol (PGS agar) [8]. This type of killing is referred to as ‘fast killing’. In contrast, when the bacteria are grown on a less rich medium, it takes several days for *C. elegans* to die [9]. This type of killing is referred to as ‘slow killing’. Fast killing is thought to be mediated by diffusible toxins because exudates of PA14 grown on PGS agar are sufficient to kill *C. elegans*; worms need not be in the presence of live PA14 in order to be killed. In contrast, slow killing requires bacterial growth in the worm gut to effect pathogenesis. Experiments in mice have demonstrated that the fast killing toxin-based model is relevant in plant and mammalian pathogenesis because mutants that are defective in fast killing are also defective in *Arabidopsis thaliana* and murine PA14 infection models [8].

In this study we focus on identification of the toxins responsible for fast killing. In a previously published screen for PA14 mutants that exhibited reduced levels of fast killing, several isolates displayed reduced levels of the blue-green phenazine pigment pyocyanin [8]. Phenazines are a class of tricyclic aromatic molecules produced by *P. aeruginosa* and several other Gram-negative and Gram-positive bacteria [10], [11], [12]. Some phenazines, especially pyocyanin, have been shown to act as toxins against bacteria, fungi, or mammals as a consequence of their redox activities [12], [13], [14], [15].

Only a subset of the previously identified *C. elegans* fast killing-deficient PA14 mutants were found to produce less pyocyanin than wild-type [8], suggesting that other phenazines or a different class of molecules are involved. Moreover, phenazine toxicity has not been demonstrated directly in *C. elegans* [8]. In order to better understand the mechanisms of *P. aeruginosa* PA14 toxicity, we sought to identify the toxin molecules produced by PA14 that kill *C. elegans*. We demonstrate that three of the phenazines produced by PA14 can rapidly kill *C. elegans*: phenazine-1-carboxylic acid kills *C. elegans* at acidic pH; pyocyanin, a product of phenazine-1-carboxylic acid, kills *C. elegans* at neutral or basic pH; 1-hydroxyphenazine, a second product of phenazine-1-carboxylic acid, kills *C. elegans* in a pH-independent manner. We also show that under the conditions of the fast killing assay phenazine-1-carboxylic acid, not pyocyanin, is the primary toxin responsible for the rapid death of *C. elegans* in the presence of PA14.

## Results

### Phenazine production is essential for killing of *C. elegans* by *P. aeruginosa* PA14

**Figure 1:**
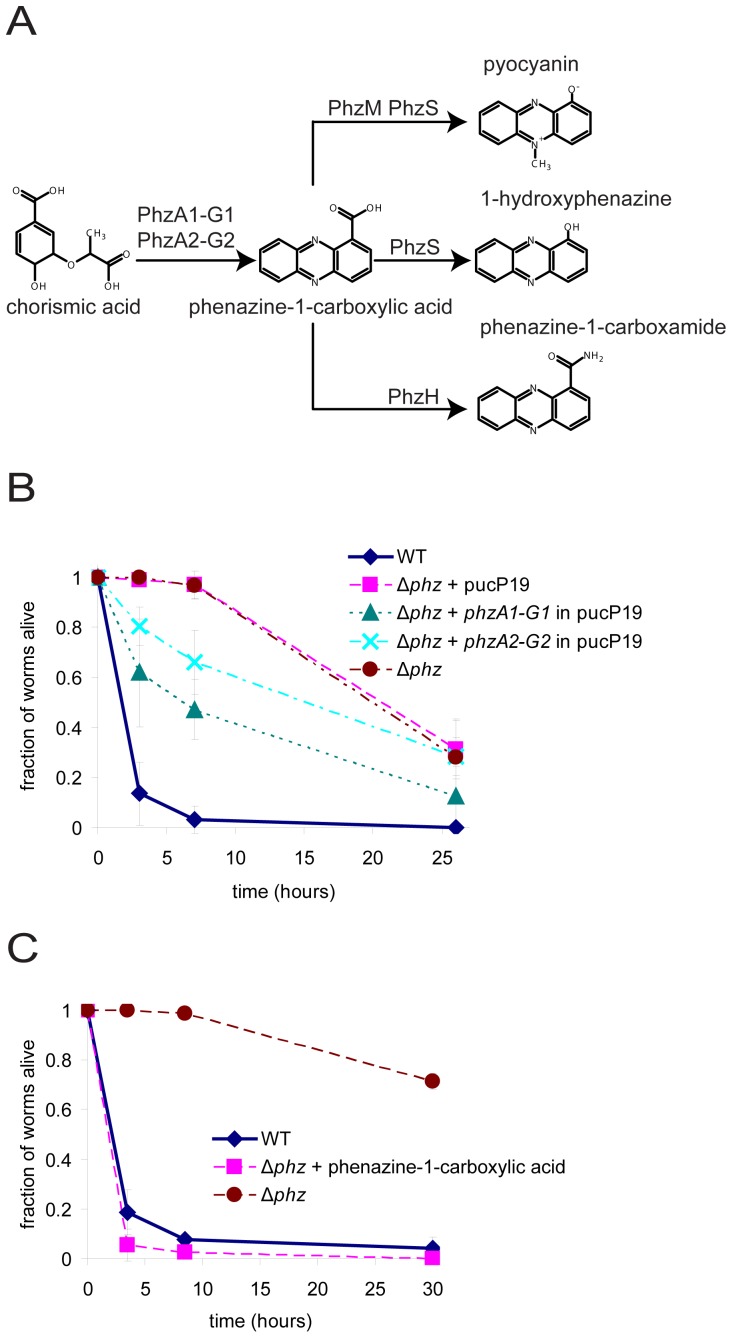
Phenazine synthesis by *P. aeruginosa* is essential for killing *C. elegans*. (A) Phenazine synthesis pathway of *P. aeruginosa*. (B) Killing of wild-type *P. aeruginosa* PA14 and Δ*phz* mutant and partial complementation of Δ*phz* killing with plasmids containing either the *phzA1-G1* or *phzA2-G2* operon. (C) Complementation of killing in the Δ*phz* mutant by addition of synthetic phenazine-1-carboxylic acid (100 µg/mL) to the agar medium prior to plating and growth of the bacteria.

As described above, among the previously isolated PA14 mutants that are deficient in toxin-mediated killing of *C. elegans* on PGS agar, those with the largest reduction in toxicity were found to produce less of the phenazine pyocyanin than wild-type PA14 [8]. Pyocyanin is one of at least four phenazines that are produced by wild-type PA14 [10], [16], [17] (Figure 1A). Phenazine-1-carboxylic acid, the precursor of all other phenazines produced by *P. aeruginosa*, is synthesized from chorismate by genes constituting the redundant *phzA1-G1* and *phzA2-G2* operons, each of which encodes a full set of functional phenazine-1-carboxylic acid biosynthetic enzymes [17]. Phenazine-1-carboxylic acid can be modified by other enzymes to make 1-hydroxyphenazine, phenazine-1-carboxamide, or pyocyanin [17].

To determine the importance of phenazines in the pathogenesis of an established *C. elegans* model host system, we tested the killing ability of a PA14 mutant that does not produce any phenazines (Δ*phz*). The Δ*phz* mutant is missing both the *phzA1-G1* operon and the *phzA2-G2* operon, precluding the production of phenazine-1-carboxylic acid as well as the other phenazines [16]. Lack of phenazine production by the Δ*phz* mutant was confirmed by metabolite profiling (Table 1). Wild-type or Δ*phz* mutant PA14 bacteria were spread on PGS agar plates and allowed to grow for 24 hours at 37°C followed by 24 hours at 23°C. L4 stage worms were then placed on the agar. Worms were scored as live or dead based on whether or not movement could be elicited by tapping their heads gently with a thin wire. We found that the Δ*phz* mutant is severely compromised in its ability to kill *C. elegans* compared to wild-type PA14 (Figure 1B). Transformation of Δ*phz* with a multicopy plasmid containing either the *phzA1-G1* operon or the *phzA2-G2* operon partially complemented the killing-deficient phenotype (Figure 1B). Chemical complementation of the Δ*phz* phenotype by the addition of 100 µg/mL of synthetic phenazine-1-carboxylic acid to the agar prior to bacterial growth restored the nematode-killing phenotype (Figure 1C), suggesting that phenazine-1-carboxylic acid or another phenazine derived from phenazine-1-carboxylic acid could be a toxin involved in PA14-mediated killing of *C. elegans*. Alternatively, phenazine-1-carboxylic acid could function indirectly to regulate the production of a toxin that kills *C. elegans*.

**Table 1:** Levels of phenazines in agar plated with PA14 mutants (µg/mL).

	phenazine-1-carboxylic acid	1-hydroxyphenazine	pyocyanin	phenazine-1-carboxamide*
Wild-type	52.71±8.41	1.36±0.11	2.38±0.28	<1
Δ*phz*	nd	nd	nd	nd
*phzH*	58.6±11.25	1.43±0.19	3.00±0.87	<1
*phzM*	70.19±6.93	1.93±0.08	nd	2.09±0.16
*phzS*	46.32±1.47	nd	nd	<1

nd = not detected.

* phenazine-1-carboxamide was detected in all strains except Δ*phz*. However, levels were below the quantifiable detection limit of 1 µg/mL for wild-type, *phzH*, and *phzS*.

### Phenazine-1-carboxylic acid and 1-hydroxyphenazine are toxic to *C. elegans*

**Figure 2:**
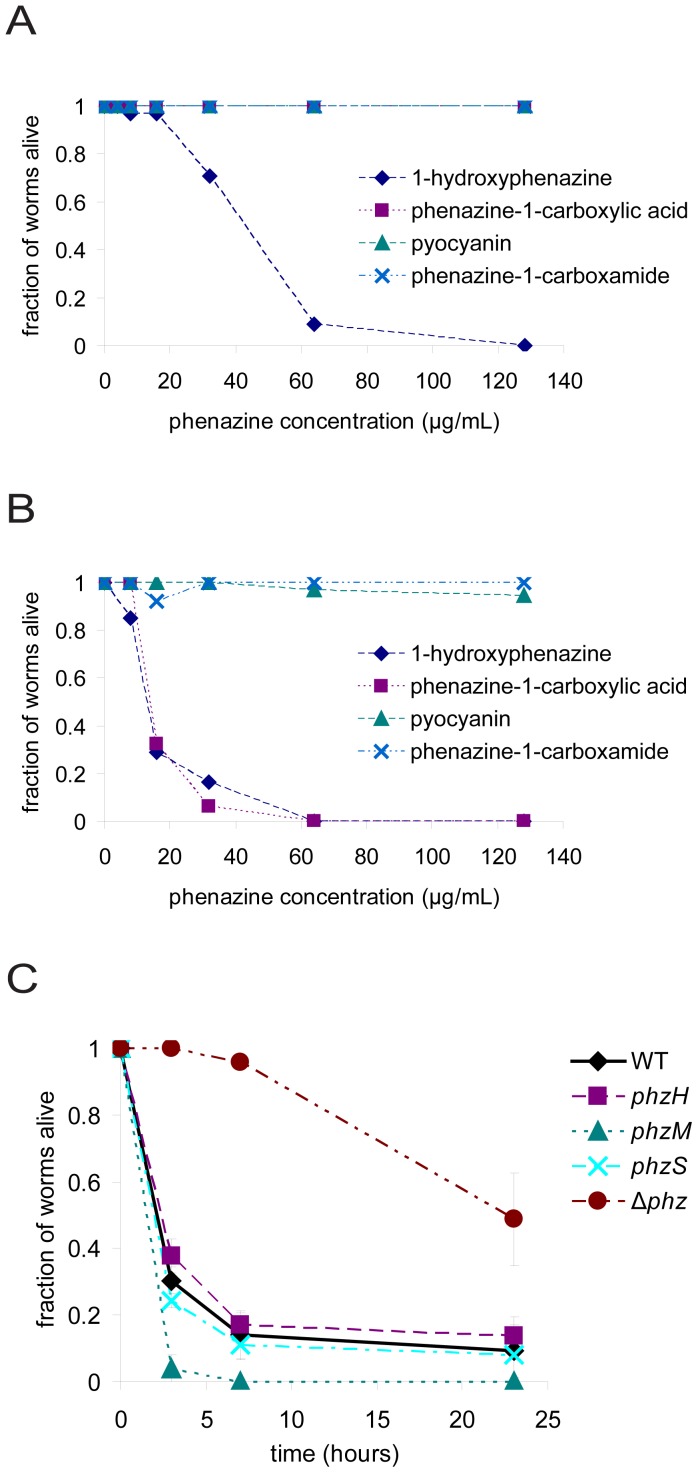
phenazine-1-carboxylic acid and 1-hydroxyphenazine are toxic to *C. elegans*. (A) Killing of *C. elegans* after four hours of exposure to synthetic phenazines (4, 8, 16, 32, 64, and 128 µg/mL final concentrations) added to naive PGS agar plates. Data points for phenazine-1-carboxylic acid, pyocyanin, and phenazine-1-carboxamide are overlapping. (B) Killing of *C. elegans* after four hours of exposure to synthetic phenazines added to PGS agar plates after growth of Δ*phz* bacteria. (C) Killing of *C. elegans* by *P. aeruginosa* PA14 mutants in the phenazine synthesis pathway.

To determine if phenazines are sufficient to kill worms, we tested the killing activities of synthetic phenazines directly by adding them to PGS agar in the absence of bacteria. Under these conditions, 1-hydroxyphenazine killed worms at concentrations above 16 µg/mL with kinetics similar to a typical fast killing assay with PA14, whereas phenazine-1-carboxylic acid, pyocyanin, and phenazine-1-carboxamide did not kill worms on a relevant time scale, even at much higher concentrations (Figures 2A, S1A). To account for potential synergistic effects of phenazines with other metabolites produced by PA14, we tested the killing activities of synthetic phenazines added to PGS agar after growth of a lawn of Δ*phz* bacteria. After growth of the lawn, the bacteria were scraped off the agar, the agar was melted in a microwave, phenazines were mixed in at several concentrations, and the agar was allowed to cool prior to introduction of the worms. We refer to the melted and cooled agar upon which the Δ*phz* bacteria had been grown as “Δ*phz* agar”. Similarly to the data shown in Figure 2A, when 1-hydroxyphenazine was added to Δ*phz* agar it killed worms rapidly at concentrations above 16 µg/mL, whereas pyocyanin and phenazine-1-carboxamide killed worms poorly, even at much higher concentrations (Figures 2B, S2B). Interestingly, contrary to its activity on naive PGS agar, on Δ*phz* agar phenazine-1-carboxylic acid also killed worms at concentrations above 16 µg/mL. These data, together with the observation that 1-hydroxyphenazine appeared to be somewhat more toxic to worms when added to Δ*phz* agar than when added to naive PGS agar (compare Figures 2A and 2B), suggested that the Δ*phz* strain produces a factor or factors that enhance the toxicities of 1-hydroxyphenazine and phenazine-1-carboxylic acid.

The data in Figure 2B showing that both 1-hydroxyphenazine and phenazine-1-carboxylic acid kill *C. elegans* when added to Δ*phz* agar indicated that either or both of these phenazines could be responsible for PA14-mediated intoxication of *C. elegans* in the fast killing assay. In considering the relative contributions to nematode death of phenazine-1-carboxylic acid and 1-hydroxyphenazine produced by PA14, we used metabolite profiling to determine the levels of phenazine-1-carboxylic acid and 1-hydroxyphenazine in PGS agar following growth of wild-type PA14. We found that the amount of phenazine-1-carboxylic acid (53 µg/mL) was greater than the level required for killing worms, whereas the amount of 1-hydroxyphenazine (1.4 µg/mL) was insufficient to kill worms to a significant degree (Table 1). These observations suggested that phenazine-1-carboxylic acid is at least partially responsible for nematode killing under the conditions of our intoxication assay.

To further investigate which phenazines are responsible for worm killing, we tested the killing abilities of PA14 mutants that are unable to synthesize pyocyanin, 1-hydroxyphenazine, or phenazine-1-carboxamide. A *phzM* mutant, which does not produce pyocyanin, killed worms more rapidly than did wild-type PA14 (Figure 2C). A *phzS* mutant, which does not produce pyocyanin or 1-hydroxyphenazine [17], [18], [19], did not show a significant difference in killing from wild-type. A *phzH* mutant, which is deficient in production of phenazine-1-carboxamide, also did not show a significant difference in killing from wild-type. The lack of production of phenazines in these mutants according to the previously described biosynthetic pathways shown in Figure 1A was confirmed by metabolite profiling (Table 1). These data, in combination with the fact that synthetic pyocyanin and phenazine-1-carboxamide do not kill worms at the concentrations they are produced under the conditions of our assay (Figure 2B, Table 1), are consistent with the conclusion that pyocyanin and phenazine-1-carboxamide do not play a significant role in fast killing. Moreover, the facts that the *phzS* mutant retains the ability to kill and that the level of 1-hydroxyphenazine produced by wild-type PA14 is insufficient to kill to a significant degree, are consistent with the conclusion that 1-hydroxyphenazine is either not necessary for killing or that it is one of multiple factors that cooperate to kill worms. We also observed that the *phzM* mutant, which kills more rapidly than wild-type PA14, produced 34% more phenazine-1-carboxylic acid than wild-type (70 µg/mL vs. 53 µg/mL; Table 1), which was the greatest amount of phenazine-1-carboxylic acid produced among the strains tested. Levels of phenazine-1-carboxylic acid were also slightly elevated with the *phzH* mutant (59 µg/mL). In contrast, the *phzS* mutant showed slightly depressed levels of phenazine-1-carboxylic acid (46 µg/mL). These data are consistent with the hypothesis that worm death requires phenazine-1-carboxylic acid. Furthermore, we observed no killing of nematodes on naive PGS agar that was supplemented with all four synthetic phenazines at concentrations comparable to those produced by wild-type PA14 (data not shown), indicating that toxicity is not induced by the combination of phenazines in the absence of other factors. These data further support the conclusion that pyocyanin and phenazine-1-carboxamide are unlikely to play major roles in worm killing under the conditions tested.

### Toxicity of phenazine-1-carboxylic acid is pH dependent

**Figure 3:**
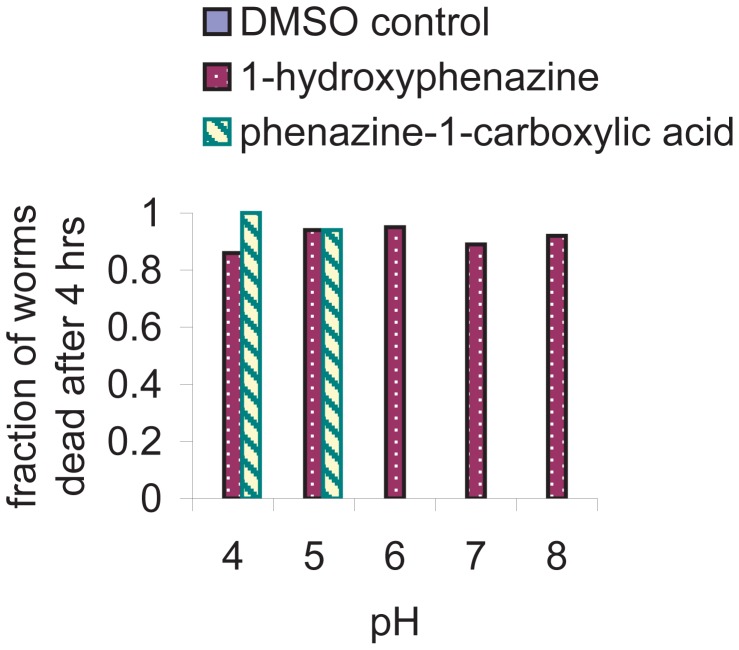
Toxicity of phenazine-1-carboxylic acid is pH dependent. Nematode death after exposure to 100 µg/mL of phenazine-1-carboxylic acid or 1-hydroxyphenazine in PGS agar plates buffered at pH 4 (50 mM sodium acetate), 5 (50 mM sodium citrate), 6 (50 mM potassium phosphate), 7 (50 mM potassium phosphate), or 8 (50 mM potassium phosphate). There is no observable killing by phenazine-1-carboxylic acid at pH 6, 7, or 8, or by DMSO without phenazines under any of the buffer conditions.

The data in Figure 2 suggested that phenazine-1-carboxylic acid is most likely the primary phenazine toxin responsible for nematode killing. Because phenazine-1-carboxylic acid killed worms when mixed with Δ*phz* agar but not when mixed with naive agar, we reasoned that at least one additional factor provided by the bacteria is necessary for the toxicity of phenazine-1-carboxylic acid. In our attempts to identify this factor, we took into consideration precedents in the literature for the pH-dependence of the activity of bacterial toxins [20], [21]. We found that the pH of PGS agar drops from approximately 6 prior to bacterial growth to between 4 and 4.5 after growth of PA14. We tested the killing activities of phenazine-1-carboxylic acid and 1-hydroxyphenazine under different buffer conditions and found that killing by phenazine-1-carboxylic acid (at 100 µg/mL) was strongly pH dependent, with low pH supporting killing and neutral or higher pH preventing killing (Figure 3). In contrast, 1-hydroxyphenazine did not show pH-dependent toxicity at the same concentration. DMSO, the solvent for the phenazine solutions, did not kill worms under any of the buffer conditions tested, demonstrating that the worms are not dying only as a consequence of exposure to the low pH buffer. These observations explained why phenazine-1-carboxylic acid failed to kill worms when added to naive agar (pH 6), and considered together with the genetic and metabolite profiling data, suggest that phenazine-1-carboxylic acid is the primary toxin responsible for PA14-mediated killing of *C. elegans* under fast killing assay conditions.

### Toxicity of pyocyanin is also pH dependent

**Figure 4:**
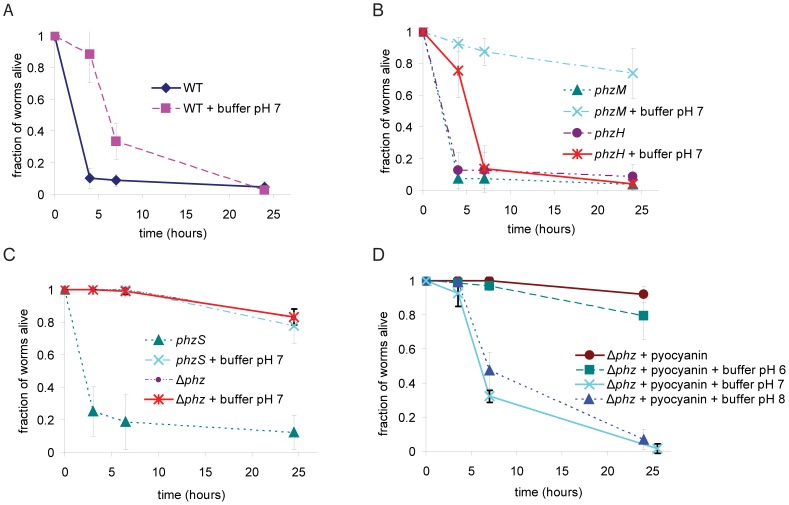
Toxicity of pyocyanin is pH dependent. (A, B, C) Nematode death on PGS agar with wild-type PA14, *phzM*, *phzH*, *phzS*, and Δ*phz*. After bacterial growth, agar was melted and potassium phosphate pH 7 (100 mM final concentration) or equal volume of water was added. Worms were added after the agar had cooled and solidified. (D) Toxicity of exogenously added pyocyanin (10 µg/mL) in Δ*phz* agar raised to pH 6, 7, or 8 with 100 mM potassium phosphate. Pyocyanin and buffer were added after bacterial growth.

If phenazine-1-carboxylic acid is the primary toxic agent and if phenazine-1-carboxylic acid is only active at low pH, we reasoned that buffering the agar media to pH≥6 after growth of PA14 would block toxin-mediated killing activity of the PA14 agar. Although we observed a delay in killing when we raised the pH of the media to 7 with potassium phosphate, a substantial fraction of the worms died within seven hours (Figure 4A). When we performed the same experiment with a *phzM* or *phzS* strain, we found that killing on the relevant time scale was abrogated (Figure 4B & C). The phenotype of the *phzH* mutant was indistinguishable from wild-type under these conditions. Similar results were obtained when the pH was raised to 8 with Tris HCl, demonstrating that the phenomenon is not specific to potassium phosphate (Figure S2). Because both *phzM* and *phzS* are required for the synthesis of pyocyanin and *phzH* is not, these data suggested that pyocyanin might be toxic to worms at pH 7 or pH 8. We tested the toxicity of pyocyanin by observing worm survival on PGS agar with synthetic pyocyanin (10 µg/mL) and buffer added after growth of Δ*phz* (Figure 4D). Addition of pyocyanin caused worm death at pH 7 and pH 8, but not at pH 6. Moreover, the kinetics of worm death at pH 7 and 8 were similar to those observed with media on which wild-type PA14 were grown and subsequently buffered at pH 7, with the toxic effects not evident until the 7-hour time point. Surprisingly, when we exposed worms to pyocyanin (10 µg/mL) at pH 8 (50 mM potassium phosphate) on PGS agar plates in the absence of bacteria, we observed no worm death within 7 hours (data not shown), suggesting that there is an unknown non-phenazine product of *P. aeruginosa* that sensitizes *C. elegans* to pyocyanin. Together, these data indicate that three of four known phenazines produced by *P. aeruginosa* strain PA14 are toxic to *C. elegans*. Toxicity of the phenazines, however, varies depending on the pH of the media as well as the presence of other factors.

## Discussion

Pathogenesis of *P. aeruginosa* can occur through a variety of mechanisms including the production and excretion of toxins. In this study we identified three phenazine toxins produced by *P. aeruginosa* PA14 that can kill *C. elegans* under different environmental conditions. We showed that under the conditions of our assay phenazine-1-carboxylic acid is the predominant toxic phenazine produced by *P. aeruginosa* PA14 that kills *C. elegans*. Our work suggests that phenazine-1-carboxylic acid should be further studied as a potentially important contributor to toxin-mediated pathogenesis in other metazoan hosts besides *C. elegans*, including mammals.

We found that the toxicity of phenazine-1-carboxylic acid to *C. elegans* requires an acidic environment. Acidic conditions are not uncommon, for example in wounds [22], [23], in the gut [24], [25], and intracellularly within lysosomes [23], [26] and secretory granules [27]. Phenazine-1-carboxylic acid may act as a toxin in a variety of circumstances in those locations. The pH dependence of phenazine-1-carboxylic acid toxicity may be due to the protonation state of its carboxyl group, for which the pK_a_ is 4.2 [28]. This hypothesis suggests that the uncharged acid species may be toxic and the negatively charged carboxylate benign, as has been observed for the antimicrobial activity of phenazine-1-carboxylic acid against *Bacillus cereus* and the fungus *Gaeumannomyces graminis* var. *tritici* [28]. Because the cytosol is buffered near neutral pH, we suspect that either the toxic effects of phenazine-1-carboxylic acid are occurring extracellularly or that the charge state affects the permeability of phenazine-1-carboxylic acid through the membrane. Neutral molecules typically traverse membranes more easily than charged molecules. After the uncharged phenazine-1-carboxylic acid species has diffused through the membrane, the neutral environment of the cytoplasm would result in its deprotonation. In its negatively charged carboxylate form it might be unable to diffuse out of the cell, resulting in its toxic accumulation within the cell. Phenazine-1-carboxamide, which differs from phenazine-1-carboxylic acid by only an amide group in place of the carboxyl group, is not toxic to *C. elegans* in our assay. The amide and the carboxyl groups should both be uncharged at acidic pH where phenazine-1-carboxylic acid is toxic and phenazine-1-carboxamide is not. Although it is possible that the chemical difference between these phenazines could affect their behavior due to other properties than their difference in pK_a_, the difference in toxicity of these two species suggests that charge state is not the sole determinant of phenazine toxicity.

The zwitterionic species of pyocyanin is the predominant form at pH 7 and 8, and the net neutral charge may facilitate its traversal through the membrane as well [15]. The pK_a_ of pyocyanin is 4.9 [29], which does not explain why pyocyanin is ineffective at killing nematodes at pH 6, where the zwitterionic form should still predominate. However, as shown in Figure 4, unlike the killing activities of phenazine-1-carboxylic acid and 1-hydroxyphenazine, the killing activity of pyocyanin appears to be dependent on a non-phenazine factor produced by *P. aeruginosa*. It is possible that the oxidation state of pyocyanin is altered by components of *P. aeruginosa* exudates on PGS agar. Phenazines, including pyocyanin, exert toxic effects in a variety of mammalian tissues through the generation of reactive oxygen species [8], [12], [13], [15], [30], [31]. Oxidized and reduced phenazines also have different hydrophobicities [12], which can affect their ability to permeate membranes or remain in aqueous solution. The oxidation state of pyocyanin has been shown to change as a function of cell density in PA14 liquid culture [32], and changes in pH also affect its redox potential [33]. The difference in pyocyanin toxicity to nematodes in the presence and absence of PA14 exudates could be due to differences in the oxidation states of pyocyanin. It is also possible that the pH dependence of pyocyanin-mediated killing is due to pH-dependent activation of the accessory factor(s) and not of pyocyanin itself. Given the importance of pyocyanin as a toxin in a variety of systems, it would be valuable to identify these accessory factors and determine if they play a role in pyocyanin toxicity in other hosts.

Studies have demonstrated that 5-methyl-phenazine-1-carboxylic acid (5MPCA), another phenazine produced by *P. aeruginosa*, is toxic to the fungus *C. albicans* [18], [19]. 5MPCA is an intermediate in pyocyanin synthesis that is produced by PhzM acting on phenazine-1-carboxylic acid. Given that the *phzM* strain is the most toxic in our assay, and that the *phzS* strain, which results in the accumulation of 5MPCA, is no more toxic than wild-type, we think it is unlikely that 5MPCA produced by PA14 is a toxic species to *C. elegans* under the conditions of our assay.

CF patients are highly susceptible to lung infections by *P. aeruginosa* [1], [2]. CF results in abnormal hyperacidification of organelles of epithelial cells in the respiratory pathway as well as increased acidity of airway surface liquid as compared with healthy individuals [34]. Hyperacidification of the lung airway surface liquid reduces its antimicrobial effects in a porcine CF model [35] and has been speculated to have other effects on the CF lung, including tissue damage, inflammation, and thickening of the mucus [34]. Increased acidity could also lead to enhanced toxicity of phenazine-1-carboxylic acid in the *P. aeruginosa* infected CF lung.

The concentrations of pyocyanin we determined that are active against nematodes are within the concentrations observed in human CF sputum and that have been shown to have physiological activity [36], [37], [38], [39], [40], [41]. In wild-type PA14 grown on PGS agar, we detected 2.38 µg/mL (11.3 µM) pyocyanin. Pyocyanin has been detected at concentrations of up to 27.3 µg/mL (130 µM) in the sol phase of CF sputum [37]. Interestingly, a recent study showed that pyocyanin concentrations in sputum from CF patients correlated with the degree of severity of the disease, ranging from 7.7 µM in unobstructed airways to 46.8 µM in severely obstructed airways [36].

Phenazine-1-carboxylic acid concentrations also correlated with impairment of lung function in CF, with concentrations ranging from 5.2 µM in unobstructed airways to 39.9 µM in severely obstructed airways [36]. The concentrations we detected in PGS agar were considerably higher, at 52.7 µg/mL (235 µM), but we demonstrated that phenazine-1-carboxylic acid is toxic to nematodes at concentrations as low as 16 µg/mL (71.4 µM). Although this is still higher than concentrations observed in CF lungs, it is possible that worms are intrinsically more resistant to phenazine-1-carboxylic acid than mammalian cells.

*Pseudomonas* infections are also common in wounds, which exhibit a variety of different pH conditions [22], [23]. During inflammation, the pH of acute wounds falls below 6, a level at which phenazine-1-carboxylic acid could exert its toxic effects. During early stages of healing, the pH increases to between 7 and 8, where phenazine-1-carboxylic acid might be ineffective as a toxin. In that pH range pyocyanin could act as a toxin. Indeed, pyocyanin has been shown to inhibit wound repair and induce cellular senescence in an *in vitro* model of wound healing [41].

In agreement with previous observations [8], [9], we observed that L4 worms were more susceptible to killing by phenazines than adult animals. We also noticed that older L4 worms were killed more quickly than younger L4 worms (B. Cezairliyan and R. Feinbaum, unpublished observations). We suspect that proximity to the L4 to adult molt plays a role in the susceptibility of L4 worms. Perhaps worms are most susceptible when they are first exposed to phenazines during the molt, when phenazines might be able to most easily due to the shedding of the cuticle. Earlier exposure to phenazines might allow worms time to detoxify them before reaching their most susceptible stage. It is also possible that specific gene expression patterns unrelated to toxin permeability during the L4 to adult molt are the cause of increased susceptibility. Identification of genes that reduce susceptibility to phenazines at this transition or that alter the developmental stage at which worms are susceptible to phenazines should help to elucidate the cause of the stage-dependent susceptibility of *C. elegans* to phenazines.

An understanding of the regulation of toxin production in *P. aeruginosa* is critical for understanding its interactions with its hosts. Phenazine biosynthesis in *P. aeruginosa* is dependent on a variety of environmental factors in the media and is also largely dependent on cell density via quorum sensing signals [12], [13], [42], [43], [44], [45], [46]. In *P. aeruginosa*, the genes that are responsible for producing phenazine-1-carboxylic acid are present in duplicate in two differently-regulated operons in the genome. The genes that regulate production of other phenazines, however, are transcribed independently of one another and of the *phzA1-G1* and *phzA2-G2* operons, thus allowing for transcriptional regulation of the production of particular phenazines depending on the environmental stimuli [17], [46], [47]. Many phenazine-producing bacteria make more than one type of phenazine [11]. pH has been shown to influence the production of phenazine-1-carboxylic acid in *Pseudomonas fluorescens* and phenazine-1-carboxamide in *Pseudomonas chlororaphis* [48], [49]. Moreover, *P. aeruginosa* grown under different conditions can produce other low molecular weight toxins that are lethal to *C. elegans*, including cyanide [5], [50] and quinolone [4]. Our work suggests that one of the purposes that could be served by the diversity of phenazines is to have an armamentarium of molecules available that are active under different environmental conditions.

Knowledge of the toxins that are effective in our nematode killing assay will allow for a better understanding of phenazine toxicity in *C. elegans* and in other hosts. Although the mutants identified in the screen for toxin-killing deficiency in PA14 were also reduced in pathogenesis in a mouse thermal injury model and an *Arabidopsis* leaf infiltration model [8], it is still unclear how the nematode intoxication assay serves as a proxy for the mammalian and plant models, which are based on infection and colonization by live bacteria. It will be important to determine if the same phenazine toxins that we have identified in the killing of *C. elegans* play a role in the murine or plant models. If the toxicity in mammals is similarly based on phenazines, *C. elegans* will continue to serve as a useful tool in the identification of pathogenesis mechanisms of *P. aeruginosa* in mammals and plants. Host pathways in *C. elegans* can be probed genetically to identify mechanisms by which phenazine-1-carboxylic acid acts to kill the worm and whether the three toxic phenazines exert their lethal effects in similar ways in the host. A combination of proteomics, gene expression, and mutant analysis should help to shed light on toxicity mechanisms of phenazines in the host and the ways by which their production is regulated in the pathogen.

## Materials and Methods

### Media and chemicals

Worms were maintained on lawns of *E. coli* OP50 on nematode growth medium agar (NGM) plates prior to killing assays. Killing assays were performed on PGS agar (1% bacto-peptone, 1% glucose, 1% NaCl, 150 mM sorbitol, 1.7% bacto-agar) as described [8]. 1-hydroxyphenazine was purchased from TCI America (H0289), pyocyanin from Cayman Chemical Company (10009594), phenazine-1-carboxylic acid from Apollo Scientific (OR01490), and phenazine-1-carboxamide from Princeton Biomolecular Research (PBMR030086). All phenazine stocks were dissolved in DMSO.

### Bacterial and worm strains

The PA14 Δ*phz* mutant strain lacking both the *phzA1-G1* and *phzA2-G2* operons was kindly provided by Lars Dietrich and Dianne Newman [16]. Other PA14 mutant strains were obtained from a non-redundant transposon insertion library [51]: *phzM* (mutant ID 40343), *phzH* (39981), *phzS* (44099). All transposon insertion strains were sequenced to confirm the location of the transposon. The *phzM* and *phzS* genes are on opposite sides of the *phzA1-G1* operon. *phzM and phzS* are coregulated to some degree with *phzA1-G1*, but they are part of separate transcriptional units not containing other genes [17], [46]. Thus, the transposons in *phzM* and *phzS* are unlikely to exert polar effects.The *phzH* gene is elsewhere in the genome and is not known to be cotranscribed with other genes [17], [31]. *C. elegans* strain Bristol N2 [52] was used for killing assays.

### *C. elegans* killing assays

Killing assays were performed as previously described [8]. In order to have fourth larval stage (L4) worms for killing assays, 10 gravid worms were picked to a 60 mm plate with NGM agar and a lawn of *E. coli* OP50 as a food source. Gravid worms were kept on plates for 16 hours at 15°C, after which they were removed. Plates were returned to 15°C for 8 hours, after which they were transferred to 20°C. Eggs hatched and grew to L4 stage approximately 36 hours after transfer. Proper staging of L4 worms was critical to the reproducibility of the assay, as worms that were younger or older were killed less quickly than L4 stage worms.

Killing agar plates were prepared by spreading 5 µL of overnight culture of PA14 in LB on a 35 mm petri plate containing 4 mL of PGS agar (1% bacto-peptone, 1% glucose, 1% NaCl, 150 mM sorbitol, 1.7% bacto-agar). Plates were incubated for 24 hours at 37°C and then transferred to 23°C for 24 hours. L4 stage worms were put on the plates, which remained at room temperature until the completion of the assay. Worms were scored as live or dead based on movement elicited by tapping their heads gently with a thin wire.

To mix the agars of plates seeded with different PA14 strains, bacteria were scraped off the surface of the agar with a cell scraper after which the agar was melted by heating in a microwave. The hot agars were mixed, repoured into plates, and allowed to cool. In experiments where phenazines or buffer were added, plate agar was melted, concentrated buffer and/or phenazine stock solution (in DMSO) was added and mixed, after which plates were repoured and allowed to cool.

### Absolute quantitation of phenazines

In order to determine absolute amounts of phenazines produced by different strains, a standard curve was constructed for each of the phenazines at concentrations of 1, 4, 16, 32, and 64 µg/mL. Killing agar plates seeded with Δ*phz* were melted and doped with a range of concentrations of synthetic phenazines. Cool, dry plates were extracted with chloroform/methanol (CHCl_3_∶MeOH∶agar = 2∶1∶1). The organic layer was transferred to another vial and concentrated under a stream of nitrogen. Samples were dissolved in 200 µL of CHCl_3_ and stored at −80°C. Prior to LC-MS analysis, reconstituted samples were diluted 200 fold in CHCl_3_ and spiked with cytosporone B (csn-B) to the final concentration of 5 µM as a standard for normalization across samples. To obtain the most accurate concentrations of phenazines, samples from different strains were prepared in the same manner and at the same time as the standards. Three experimental replicates were used for each standard and bacterial sample.

LC-MS analysis was performed using an Agilent 6410 LC-ESI-QQQ instrument in multiple reaction monitoring (MRM) mode. Samples were analyzed both in the negative and positive ion modes as previously described [53]. For the LC analysis in the negative ion mode, a Gemini (Phenomenex) C18 column (5 µm, 4.6 mm×50 mm) was used together with a precolumn (C18, 3.5 µm, 2 mm×20 mm). Mobile phase A consisted of a 95/5 water/methanol mixture, and mobile phase B was made up of 60/35/5 isopropanol/methanol/water. Both A and B were supplemented with 0.1% ammonium hydroxide as solvent modifiers. For the LC analysis in the positive ion mode, a Luna (Phenomenex) C5 column (5 µm, 4.6 mm×50 mm) was used together with a precolumn (C4, 3.5 µm, 2 mm×20 mm). Mobile phases A and B, as well as the gradient, were the same as that used for the negative ion mode analysis, except that, in this case, both A and B were supplemented with 0.1% formic acid and 5 mM ammonium formate as solvent modifiers. The gradient started at 0% B for 3 min at 0.2 mL/min and then linearly increased to 100% B over the course of 8 min at 0.5 mL/min, followed by an isocratic gradient of 100% B for 4 min at 0.5 mL/min before equilibration for 5 min at 0.5 mL/min. The total analysis time, including 3 min at 0.2 mL/min, was 20 min.

MS analysis was performed with an electrospray ionization (ESI) source. The capillary voltage was set at 4000 V. The drying gas temperature was 350°C, the drying gas flow rate was 10 L/min, and the nebulizer pressure was 45 psi. For both ionization conditions, data was collected in the profile mode. Conditions for MRM experiment were optimized using the program called Optimizer (Agilent). The resulting optimized conditions for each transition used to quantitate each phenazines are listed in Supplementary Table S1. Each run was performed using a 1 µL injection of extract.

For data analysis, ions corresponding to each phenazine and csn-B were extracted from the total ion chromatograms to obtain integrated mass ion intensities (peak area; MSII) for each ion. The levels of phenazines were normalized by dividing MSII of phenazines by MSII of csn-B detected in each chromatogram to give normalized MSII (nMSII). nMSII of the standard samples was applied to construct standard curves. Finally, phenazine concentrations in the experimental samples were calculated according to the standard curves.

## Supporting Information

Figure S1 **Phenazine toxicity, replicate experiment.** (A) Killing of *C. elegans* after four hours of exposure to synthetic phenazines (4, 8, 16, 32, 64, and 128 µg/mL final concentrations) added to naive PGS agar plates. Data points for phenazine-1-carboxylic acid, pyocyanin, and phenazine-1-carboxamide are overlapping. (B) Killing of *C. elegans* after four hours of exposure to synthetic phenazines added to PGS agar plates after growth of Δ*phz* bacteria.
(TIF)

Figure S2 **Toxicity of PA14 exudates at basic pH is independent of phosphate buffer.** Nematode death on PGS agar with wild-type PA14, *phzM*, *phzH*, and *phzS*. After bacterial growth, agar was melted and Tris HCl pH 8 (100 mM final concentration) was added. Worms were added after the agar had cooled and solidified.
(TIF)

Table S1 **MRM conditions and transitions used to quantitate levels of phenazines.**
(DOC)

## References

[1] de Vrankrijker AM, Wolfs TF, van der Ent CK (2010) Challenging and emerging pathogens in cystic fibrosis. Paediatr Respir Rev 11: 246–254. 21109184 10.1016/j.prrv.2010.07.003

[2] Williams BJ, Dehnbostel J, Blackwell TS (2010) Pseudomonas aeruginosa: host defence in lung diseases. Respirology 15: 1037–1056. 20723140 10.1111/j.1440-1843.2010.01819.x

[3] Bleves S, Viarre V, Salacha R, Michel GP, Filloux A, et al (2010) Protein secretion systems in Pseudomonas aeruginosa: A wealth of pathogenic weapons. Int J Med Microbiol 300: 534–543. 20947426 10.1016/j.ijmm.2010.08.005

[4] Zaborin A, Romanowski K, Gerdes S, Holbrook C, Lepine F, et al (2009) Red death in Caenorhabditis elegans caused by Pseudomonas aeruginosa PAO1. Proc Natl Acad Sci U S A 106: 6327–6332. 19369215 10.1073/pnas.0813199106 PMC2669342

[5] Gallagher LA, Manoil C (2001) Pseudomonas aeruginosa PAO1 kills Caenorhabditis elegans by cyanide poisoning. J Bacteriol 183: 6207–6214. 11591663 10.1128/JB.183.21.6207-6214.2001 PMC100099

[6] Irazoqui JE, Urbach JM, Ausubel FM (2010) Evolution of host innate defence: insights from Caenorhabditis elegans and primitive invertebrates. Nat Rev Immunol 10: 47–58. 20029447 10.1038/nri2689 PMC2965059

[7] Marsh EK, May RC (2012) Caenorhabditis elegans, a model organism for investigating immunity. Appl Environ Microbiol 78: 2075–2081. 22286994 10.1128/AEM.07486-11 PMC3302602

[8] Mahajan-Miklos S, Tan MW, Rahme LG, Ausubel FM (1999) Molecular mechanisms of bacterial virulence elucidated using a Pseudomonas aeruginosa-Caenorhabditis elegans pathogenesis model. Cell 96: 47–56. 9989496 10.1016/s0092-8674(00)80958-7

[9] Tan MW, Mahajan-Miklos S, Ausubel FM (1999) Killing of Caenorhabditis elegans by Pseudomonas aeruginosa used to model mammalian bacterial pathogenesis. Proc Natl Acad Sci U S A 96: 715–720. 9892699 10.1073/pnas.96.2.715 PMC15202

[10] Mentel M, Ahuja EG, Mavrodi DV, Breinbauer R, Thomashow LS, et al (2009) Of two make one: the biosynthesis of phenazines. Chembiochem 10: 2295–2304. 19658148 10.1002/cbic.200900323

[11] Pierson LS 3rd, Pierson EA (2010) Metabolism and function of phenazines in bacteria: impacts on the behavior of bacteria in the environment and biotechnological processes. Appl Microbiol Biotechnol 86: 1659–1670. 20352425 10.1007/s00253-010-2509-3 PMC2858273

[12] Price-Whelan A, Dietrich LE, Newman DK (2006) Rethinking ‘secondary’ metabolism: physiological roles for phenazine antibiotics. Nat Chem Biol 2: 71–78. 16421586 10.1038/nchembio764

[13] Lau GW, Hassett DJ, Ran H, Kong F (2004) The role of pyocyanin in Pseudomonas aeruginosa infection. Trends Mol Med 10: 599–606. 15567330 10.1016/j.molmed.2004.10.002

[14] Lau GW, Ran H, Kong F, Hassett DJ, Mavrodi D (2004) Pseudomonas aeruginosa pyocyanin is critical for lung infection in mice. Infect Immun 72: 4275–4278. 15213173 10.1128/IAI.72.7.4275-4278.2004 PMC427412

[15] Liu GY, Nizet V (2009) Color me bad: microbial pigments as virulence factors. Trends Microbiol 17: 406–413. 19726196 10.1016/j.tim.2009.06.006 PMC2743764

[16] Dietrich LE, Price-Whelan A, Petersen A, Whiteley M, Newman DK (2006) The phenazine pyocyanin is a terminal signalling factor in the quorum sensing network of Pseudomonas aeruginosa. Mol Microbiol 61: 1308–1321. 16879411 10.1111/j.1365-2958.2006.05306.x

[17] Mavrodi DV, Bonsall RF, Delaney SM, Soule MJ, Phillips G, et al (2001) Functional analysis of genes for biosynthesis of pyocyanin and phenazine-1-carboxamide from Pseudomonas aeruginosa PAO1. J Bacteriol 183: 6454–6465. 11591691 10.1128/JB.183.21.6454-6465.2001 PMC100142

[18] Gibson J, Sood A, Hogan DA (2009) Pseudomonas aeruginosa-Candida albicans interactions: localization and fungal toxicity of a phenazine derivative. Appl Environ Microbiol 75: 504–513. 19011064 10.1128/AEM.01037-08 PMC2620721

[19] Morales DK, Jacobs NJ, Rajamani S, Krishnamurthy M, Cubillos-Ruiz JR, et al (2010) Antifungal mechanisms by which a novel Pseudomonas aeruginosa phenazine toxin kills Candida albicans in biofilms. Mol Microbiol 78: 1379–1392. 21143312 10.1111/j.1365-2958.2010.07414.x PMC3828654

[20] Dickerson TJ, Janda KD (2006) The use of small molecules to investigate molecular mechanisms and therapeutic targets for treatment of botulinum neurotoxin A intoxication. ACS Chem Biol 1: 359–369. 17163773 10.1021/cb600179d

[21] Wedekind JE, Trame CB, Dorywalska M, Koehl P, Raschke TM, et al (2001) Refined crystallographic structure of Pseudomonas aeruginosa exotoxin A and its implications for the molecular mechanism of toxicity. J Mol Biol 314: 823–837. 11734000 10.1006/jmbi.2001.5195

[22] Gethin G (2007) The significance of surface pH in chronic wounds. Wounds UK 3: 52–56.

[23] Schneider LA, Korber A, Grabbe S, Dissemond J (2007) Influence of pH on wound-healing: a new perspective for wound-therapy? Arch Dermatol Res 298: 413–420. 17091276 10.1007/s00403-006-0713-x

[24] Pfeiffer J, Johnson D, Nehrke K (2008) Oscillatory transepithelial H(+) flux regulates a rhythmic behavior in C. elegans. Curr Biol 18: 297–302. 18291648 10.1016/j.cub.2008.01.054 PMC2350219

[25] Evans DF, Pye G, Bramley R, Clark AG, Dyson TJ, et al (1988) Measurement of gastrointestinal pH profiles in normal ambulant human subjects. Gut 29: 1035–1041. 3410329 10.1136/gut.29.8.1035 PMC1433896

[26] Mindell JA (2012) Lysosomal acidification mechanisms. Annu Rev Physiol 74: 69–86. 22335796 10.1146/annurev-physiol-012110-142317

[27] Paroutis P, Touret N, Grinstein S (2004) The pH of the secretory pathway: measurement, determinants, and regulation. Physiology (Bethesda) 19: 207–215. 15304635 10.1152/physiol.00005.2004

[28] Brisbane PG, Janik LJ, Tate ME, Warren RF (1987) Revised structure for the phenazine antibiotic from Pseudomonas fluorescens 2–79 (NRRL B-15132). Antimicrob Agents Chemother 31: 1967–1971. 3125789 10.1128/aac.31.12.1967 PMC175836

[29] Friedheim E, Michaelis L (1931) Potentiometric study of pyocyanine. J Biol Chem 91: 355–368.

[30] Hassan HM, Fridovich I (1980) Mechanism of the antibiotic action pyocyanine. J Bacteriol 141: 156–163. 6243619 10.1128/jb.141.1.156-163.1980 PMC293551

[31] Mavrodi DV, Blankenfeldt W, Thomashow LS (2006) Phenazine compounds in fluorescent Pseudomonas spp. biosynthesis and regulation. Annu Rev Phytopathol 44: 417–445. 16719720 10.1146/annurev.phyto.44.013106.145710

[32] Price-Whelan A, Dietrich LE, Newman DK (2007) Pyocyanin alters redox homeostasis and carbon flux through central metabolic pathways in Pseudomonas aeruginosa PA14. J Bacteriol 189: 6372–6381. 17526704 10.1128/JB.00505-07 PMC1951912

[33] Wang Y, Newman DK (2008) Redox reactions of phenazine antibiotics with ferric (hydr)oxides and molecular oxygen. Environ Sci Technol 42: 2380–2386. 18504969 10.1021/es702290a PMC2778262

[34] Poschet J, Perkett E, Deretic V (2002) Hyperacidification in cystic fibrosis: links with lung disease and new prospects for treatment. Trends Mol Med 8: 512–519. 12421684 10.1016/s1471-4914(02)02414-0

[35] Pezzulo AA, Tang XX, Hoegger MJ, Alaiwa MH, Ramachandran S, et al (2012) Reduced airway surface pH impairs bacterial killing in the porcine cystic fibrosis lung. Nature 487: 109–113. 22763554 10.1038/nature11130 PMC3390761

[36] Hunter RC, Klepac-Ceraj V, Lorenzi MM, Grotzinger H, Martin TR, et al (2012) Phenazine Content in the Cystic Fibrosis Respiratory Tract Negatively Correlates with Lung Function and Microbial Complexity. Am J Respir Cell Mol Biol Epub ahead of print. 10.1165/rcmb.2012-0088OC 22865623

[37] Wilson R, Sykes DA, Watson D, Rutman A, Taylor GW, et al (1988) Measurement of Pseudomonas aeruginosa phenazine pigments in sputum and assessment of their contribution to sputum sol toxicity for respiratory epithelium. Infect Immun 56: 2515–2517. 3137173 10.1128/iai.56.9.2515-2517.1988 PMC259599

[38] Bianchi SM, Prince LR, McPhillips K, Allen L, Marriott HM, et al (2008) Impairment of apoptotic cell engulfment by pyocyanin, a toxic metabolite of Pseudomonas aeruginosa. Am J Respir Crit Care Med 177: 35–43. 17916805 10.1164/rccm.200612-1804OC PMC7611754

[39] Muller M (2002) Pyocyanin induces oxidative stress in human endothelial cells and modulates the glutathione redox cycle. Free Radic Biol Med 33: 1527–1533. 12446210 10.1016/s0891-5849(02)01087-0

[40] O'Malley YQ, Reszka KJ, Spitz DR, Denning GM, Britigan BE (2004) Pseudomonas aeruginosa pyocyanin directly oxidizes glutathione and decreases its levels in airway epithelial cells. Am J Physiol Lung Cell Mol Physiol 287: L94–103. 15020296 10.1152/ajplung.00025.2004

[41] Muller M, Li Z, Maitz PK (2009) Pseudomonas pyocyanin inhibits wound repair by inducing premature cellular senescence: role for p38 mitogen-activated protein kinase. Burns 35: 500–508. 19286324 10.1016/j.burns.2008.11.010

[42] Deziel E, Lepine F, Milot S, He J, Mindrinos MN, et al (2004) Analysis of Pseudomonas aeruginosa 4-hydroxy-2-alkylquinolines (HAQs) reveals a role for 4-hydroxy-2-heptylquinoline in cell-to-cell communication. Proc Natl Acad Sci U S A 101: 1339–1344. 14739337 10.1073/pnas.0307694100 PMC337054

[43] Whiteley M, Lee KM, Greenberg EP (1999) Identification of genes controlled by quorum sensing in Pseudomonas aeruginosa. Proc Natl Acad Sci U S A 96: 13904–13909. 10570171 10.1073/pnas.96.24.13904 PMC24163

[44] Schuster M, Lostroh CP, Ogi T, Greenberg EP (2003) Identification, timing, and signal specificity of Pseudomonas aeruginosa quorum-controlled genes: a transcriptome analysis. J Bacteriol 185: 2066–2079. 12644476 10.1128/JB.185.7.2066-2079.2003 PMC151497

[45] Wagner VE, Bushnell D, Passador L, Brooks AI, Iglewski BH (2003) Microarray analysis of Pseudomonas aeruginosa quorum-sensing regulons: effects of growth phase and environment. J Bacteriol 185: 2080–2095. 12644477 10.1128/JB.185.7.2080-2095.2003 PMC151498

[46] Wurtzel O, Yoder-Himes DR, Han K, Dandekar AA, Edelheit S, et al (2012) The Single-Nucleotide Resolution Transcriptome of Pseudomonas aeruginosa Grown in Body Temperature. PLoS Pathog 8: e1002945. 23028334 10.1371/journal.ppat.1002945 PMC3460626

[47] Chugani SA, Whiteley M, Lee KM, D'Argenio D, Manoil C, et al (2001) QscR, a modulator of quorum-sensing signal synthesis and virulence in Pseudomonas aeruginosa. Proc Natl Acad Sci U S A 98: 2752–2757. 11226312 10.1073/pnas.051624298 PMC30211

[48] Slininger PJ, Shea-Wilbur MA (1995) Liquid-culture pH, temperature, and carbon (not nitrogen) source regulate phenazine productivity of the take-all biocontrol agent Pseudomonas fluorescens 2–79. Appl Microbiol Biotechnol 43: 794–800. 7576546 10.1007/BF02431910

[49] van Rij ET, Wesselink M, Chin AWTF, Bloemberg GV, Lugtenberg BJ (2004) Influence of environmental conditions on the production of phenazine-1-carboxamide by Pseudomonas chlororaphis PCL1391. Mol Plant Microbe Interact 17: 557–566. 15141960 10.1094/MPMI.2004.17.5.557

[50] Darby C, Cosma CL, Thomas JH, Manoil C (1999) Lethal paralysis of Caenorhabditis elegans by Pseudomonas aeruginosa. Proc Natl Acad Sci U S A 96: 15202–15207. 10611362 10.1073/pnas.96.26.15202 PMC24797

[51] Liberati NT, Urbach JM, Miyata S, Lee DG, Drenkard E, et al (2006) An ordered, nonredundant library of Pseudomonas aeruginosa strain PA14 transposon insertion mutants. Proc Natl Acad Sci U S A 103: 2833–2838. 16477005 10.1073/pnas.0511100103 PMC1413827

[52] Brenner S (1974) The genetics of Caenorhabditis elegans. Genetics 77: 71–94. 4366476 10.1093/genetics/77.1.71 PMC1213120

[53] Vinayavekhin N, Saghatelian A (2009) Regulation of alkyl-dihydrothiazole-carboxylates (ATCs) by iron and the pyochelin gene cluster in Pseudomonas aeruginosa. ACS Chem Biol 4: 617–623. 19621937 10.1021/cb900075n

