# Supplementary figures and images for: Identification of Pseudomonas aeruginosa Phenazines that Kill Caenorhabditis elegans
Source: PLoS Pathog. 2013 Jan 3;9(1):e1003101. doi: 10.1371/journal.ppat.1003101 (PMC3536714; doi:10.1371/journal.ppat.1003101)

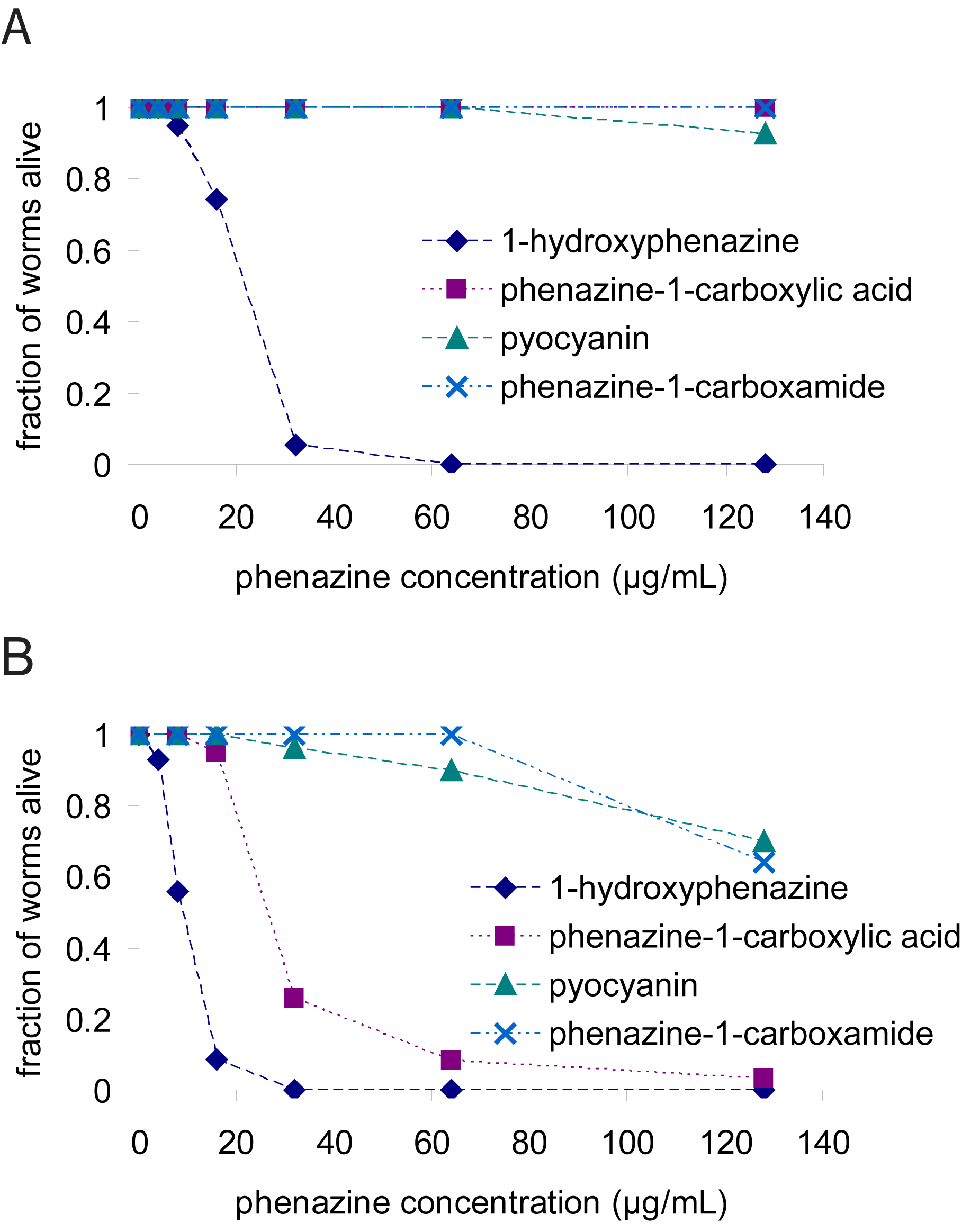

Supplement: Figure S1 — Phenazine toxicity, replicate experiment. (A) Killing of C. elegans after four hours of exposure to synthetic phenazines (4, 8, 16, 32, 64, and 128 µg/mL final concentrations) added to naive PGS agar plates. Data points for phenazine-1-carboxylic acid, pyocyanin, and phenazine-1-carboxamide are overlapping. (B) Killing of C. elegans after four hours of exposure to synthetic phenazines added to PGS agar plates after growth of Δphz bacteria. (TIF) [file ppat.1003101.s001.tif]

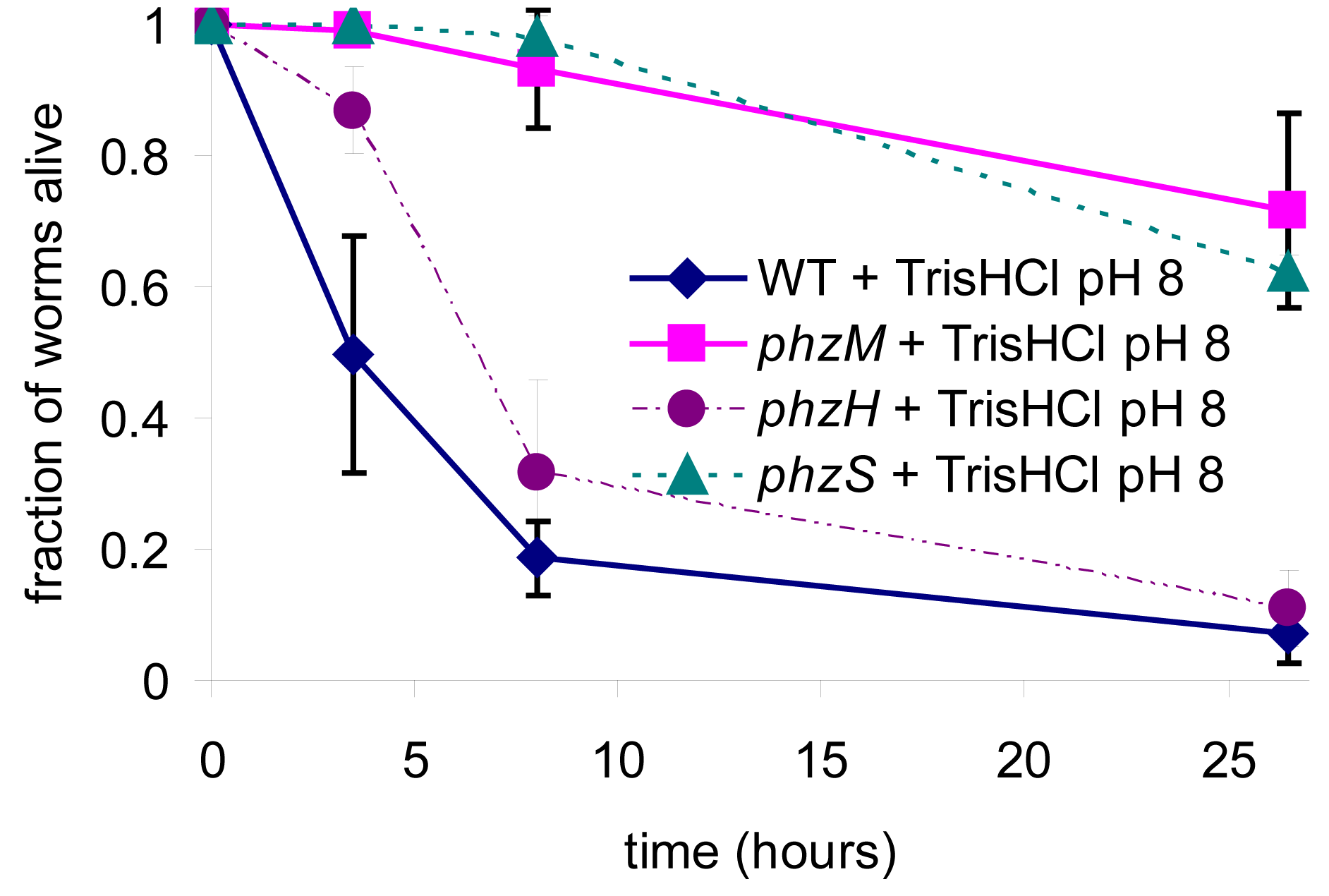

Supplement: Figure S2 — Toxicity of PA14 exudates at basic pH is independent of phosphate buffer. Nematode death on PGS agar with wild-type PA14, phzM, phzH, and phzS. After bacterial growth, agar was melted and Tris HCl pH 8 (100 mM final concentration) was added. Worms were added after the agar had cooled and solidified. (TIF) [file ppat.1003101.s002.tif]
