# Supplementary material for: Identification of Pseudomonas aeruginosa Phenazines that Kill Caenorhabditis elegans
Source: PLoS Pathog. 2013 Jan 3;9(1):e1003101. doi: 10.1371/journal.ppat.1003101 (PMC3536714; doi:10.1371/journal.ppat.1003101)
Supplement: Table S1 — MRM conditions and transitions used to quantitate levels of phenazines. (DOC) [file ppat.1003101.s003.doc]

| **Table S1: MRM conditions and transitions used to quantitate levels of phenazines** | | | | | | |
| --- | --- | --- | --- | --- | --- | --- |
| **Compounds** | **Precursor Ions (m/z)** | **Product Ions (m/z)** | **Dwell time (ms)** | **Fragmentor voltages (V)** | **Collision energy (V)** | **Used to quantitate?** |
|  |  |  |  |  |  |  |
| **Negative ion mode** | |  |  |  |  |  |
| phenazine-1-carboxylic acid | 223.1 | 275.2 | 200 | 73 | 10 | yes |
| csn-B | 321.2 | 275.2 | 200 | 108 | 17 | yes |
|  |  |  |  |  |  |  |
| **Positive ion mode** | |  |  |  |  |  |
| phenazine-1-carboxamide | 224.1 | 152.0 | 150 | 82 | 42 | yes |
| phenazine-1-carboxamide | 224.1 | 75.1 | 50 | 82 | 70 | no |
| pyocyanin | 211.1 | 183.1 | 50 | 123 | 22 | no |
| pyocyanin | 211.1 | 168.1 | 150 | 123 | 34 | yes |
| 1-hydroxyphenazine | 197.1 | 179.0 | 50 | 126 | 26 | no |
| 1-hydroxyphenazine | 197.1 | 169.1 | 150 | 126 | 26 | yes |
| csn-B | 323.2 | 277.0 | 50 | 65 | 4 | yes |
|  |  |  |  |  |  |  |
|  |  |  |  |  |  |  |
